# Filgrastim, lenograstim and pegfilgrastim in the mobilization of peripheral blood progenitor cells in patients with lymphoproliferative malignancies

**DOI:** 10.1007/s10238-014-0282-9

**Published:** 2014-04-11

**Authors:** Roberto Ria, Antonia Reale, Assunta Melaccio, Vito Racanelli, Franco Dammacco, Angelo Vacca

**Affiliations:** 1 Department of Biomedical Sciences and Human Oncology, Section of Internal Medicine and Clinical Oncology, University of Bari Medical School, Bari, Italy; 2 Department of Internal Medicine and Clinical Oncology, University of Bari Medical School, Policlinico, Piazza Giulio Cesare, 11, 70124 Bari, Italy

**Keywords:** Cell mobilization, Granulocyte colony-stimulating factor, Pegylated G-CSF, Peripheral blood progenitor cells, Recombinant human glycosylated G-CSF, Recombinant non-glycosylated G-CSF

## Abstract

Patients with lymphoproliferative disorders, candidate to autologous stem cell transplantation (ASCT), require mobilization with chemotherapy and granulocyte colony -stimulating factor (G-CSF). This study looked for differences in hematopoietic peripheral stem cells (HPSCs) mobilization in response to the three available G-CSFs, namely lenograstim, filgrastim, and pegfilgrastim. Between 2000 and 2012, 146 patients (66 M and 80 F) who underwent ASCT for multiple myeloma, non-Hodgkin’s lymphoma or Hodgkin’s lymphoma were studied. All patients received induction therapy and then a mobilization regimen with cyclophosphamide plus lenograstim, or filgrastim, or pegfilgrastim. From days 12 to 14, HPSCs were collected by two to three daily leukaphereses. Our results show that high-dose cyclophosphamide plus lenograstim achieved adequate mobilization and the collection target more quickly and with fewer leukaphereses as compared to filgrastim and pegfilgrastim. No differences between the three regimens were observed regarding toxicity and days to WBC and platelet recovery. Thus, lenograstim may represent the ideal G-CSF for PBSC mobilization in patients with lymphoproliferative diseases. Further studies are needed to confirm these results and better understand the biological bases of these differences.

## Introduction

In the current clinical practice, dose-intensive chemotherapy followed by peripheral blood stem cell transplantation (PBSCT) is frequently employed in the treatment of a variety of hematological malignancies. Granulocyte colony-stimulating factor (G-CSF), the primary regulator of granulopoiesis used to mobilize stem cells from bone marrow to peripheral blood in lymphoproliferative disorders, is primarily used for the prevention of neutropenia and reduction in its complications after chemotherapy or myelosuppressive therapy [1].

Granulocyte colony-stimulating factor (G-CSF), that has to a large extent replaced the use of granulocyte macrophage (GM)-CSF, induces the proliferation and differentiation of myeloid precursor cells, chemotaxis, respiratory burst and antigen expression of neutrophils [2, 3]. We have observed important differences using the three available G-CSFs in HPSCs mobilizing regimens.

As previously described [4], lenograstim (glycosylated rHu G-CSF) is obtained from Chinese hamster ovarian cells, which consists of 174 amino acids with 4 % glycosylation. Filgrastim (non-glycosylated Hu G-CSF) is produced using *Escherichia coli*, with a methionine group at its N-terminal end [3]. The pegylated form of non-glycosylated Hu G-CSF (pegfilgrastim) is obtained by the attachment of the polyethylene glycol (PEG) moiety, which reduces renal excretion and masks the proteolytic cleavage sites, resulting in elevated G-CSF serum levels for up to 14 days after a single injection [5]. Actually, only lenograstim is fully authorized in USA and EU for mobilization, while the other G-CSFs lack in this indication.

In some patients, the number of mobilized CD34+ cells is not sufficient to perform a successful stem cell transplantation due to bone marrow damage by neoplastic proliferation and/or chemoradiotherapy. To improve the collection of CD34+ cells, the mobilization procedure can be repeated or an alternative chemotherapy regimen can be chosen. Recently, in patients with non-Hodgkin lymphoma (NHL) or multiple myeloma (MM) with a poor yield of CD34+ cells, the new drug plerixafor (Mozobil^®^) can be administered before apheresis to increase the number of circulating CD34+ cells. Plerixafor is a derivative of bicyclam, reversible and selective antagonist of the CXCR4 chemokine receptor that acts by blocking the binding between this receptor expressed on hematopoietic stem cells and its ligand, namely the stromal cell-derived factor-1α (SDF-1α), also called CXCL12, expressed on stromal cells. Its use increases the level of functional HPCs in the peripheral blood, with long-term resettlement [6–10].

Since no data on the comparison between the three G-CSFs are available, in this study we have compared them to indentify the best mobilizer.

## Patients and methods

### Patients

A total of 146 patients (66 M and 80 F, Table 1) who underwent ASCT for MM (89 pts), NHL (46 pts) or Hodgkin lymphoma (HL, 11 pts) between 2000 and 2012 were consecutively included in this controlled, non-randomized study. Inclusion criteria were those for ASCT, namely age <70 years, serum creatinine level <200 mmol/L, cardiac ejection fraction >50 %, DLCO (diffusing capacity of the lung for carbon monoxide) >50 %, no active infection or other disease causing co-morbidity [11, 12].

**Table 1:** Patients’ characteristics

Characteristics	Total number of patients	Treated with lenograstim	Treated with filgrastim	Treated with pegfilgrastim
Numbers	146	81	31	34
Sex: M/F	66/80	29/52	19/12	18/16
Age, median (range) years	53.7 (36–64)	52.2 (36–64)	49.5 (34–60)	48.3 (38–63)
MM/NHL/HL	89/46/11	63/13/5	12/15/4	14/18/2
Status at mobilization: CR/PR/SD/PD	22/86/28/10	13/47/17/4	4/19/5/3	5/20/6/3
Patients receiving radiotherapy	12	6	2	4
Patients with bone marrow involvement	17	7	4	6
Mobilizing chemotherapy (MM/NHL/HL)				
CTX 7 g/m^2^	0/46/11	0/13/6	0/15/3	0/18/2
CTX 4 g/m^2^	74/0/0	53/0/0	10/0/0	11/0/0
CTX 3 g/m^2^	15/0/0	9/0/0	3/0/0	3/0/0

The study, which was carried out according to the Helsinki Declaration, was approved by the local ethical committee. All patients gave their informed consent.

### Treatment plan

All patients received induction therapy with 4 cycles of vincristine/adriamycin/dexamethasone (VAD—54 MM pts), 4 cycles of bortezomib/thalidomide/dexamethasone (VTD—35 MM pts), or 3 cycles of ifosfamide/epirubicin/vincristine (IEV) (LNH and LH pts), and then a mobilization regimen with cyclophosphamide 3–7 g/mq at day 0 plus glycosylated Hu G-CSF at the dose of 10 μg/kg/daily (Arm A) from day 5, or non-glycosylated rHu G-CSF (Arm B) from day 5 or pegfilgrastim (Arm C) at the dose of 6 mg at day 6. From day 12 to day 14, PBSCs were collected in two to three daily leukaphereses, and their content in CD34+ cells was evaluated by FACS analysis. If the minimum HPC target (≥4 × 10^6^/CD34+ cells/Kg bw) was not reached, after at least 1 month, Plerixafor was administered and the mobilizing procedure was repeated. This last procedure was not included in the study.

### Efficacy and toxicity

The number of CD34+ cells collected and the number of leukaphereses needed to reach their collection target were evaluated. Moreover, the number of days required to recover normal WBC and platelet counts, assessment of toxicity and the percentage of patients who achieved the collection target in a single course of mobilization (high-dose cyclophosphamide plus G-CSF, followed by three leukaphereses) were estimated.

### Statistical analysis

Data were analyzed with the SPSS, Chicago, IL, software package. All results are presented as median ±1 SD. The medians were compared with the Mann–Whitney *U* Test; *p* values <0.05 were considered significant.

## Results

A total of 81 patients (29 M and 52 F, median age 52.2 years, range 36–64) were enrolled in Arm A; 31 patients (19 M and 12 F, median age 49.5 years, range 34–60) in Arm B and 34 patients (18 M and 16 F, median age 48.3 years, range 38–63) in Arm C. At the time of mobilization, 22 patients had achieved complete remission, 86 partial remission, 28 stable disease and 10 disease progression.

### Mobilization and leukapheresis

**Fig. 1:**
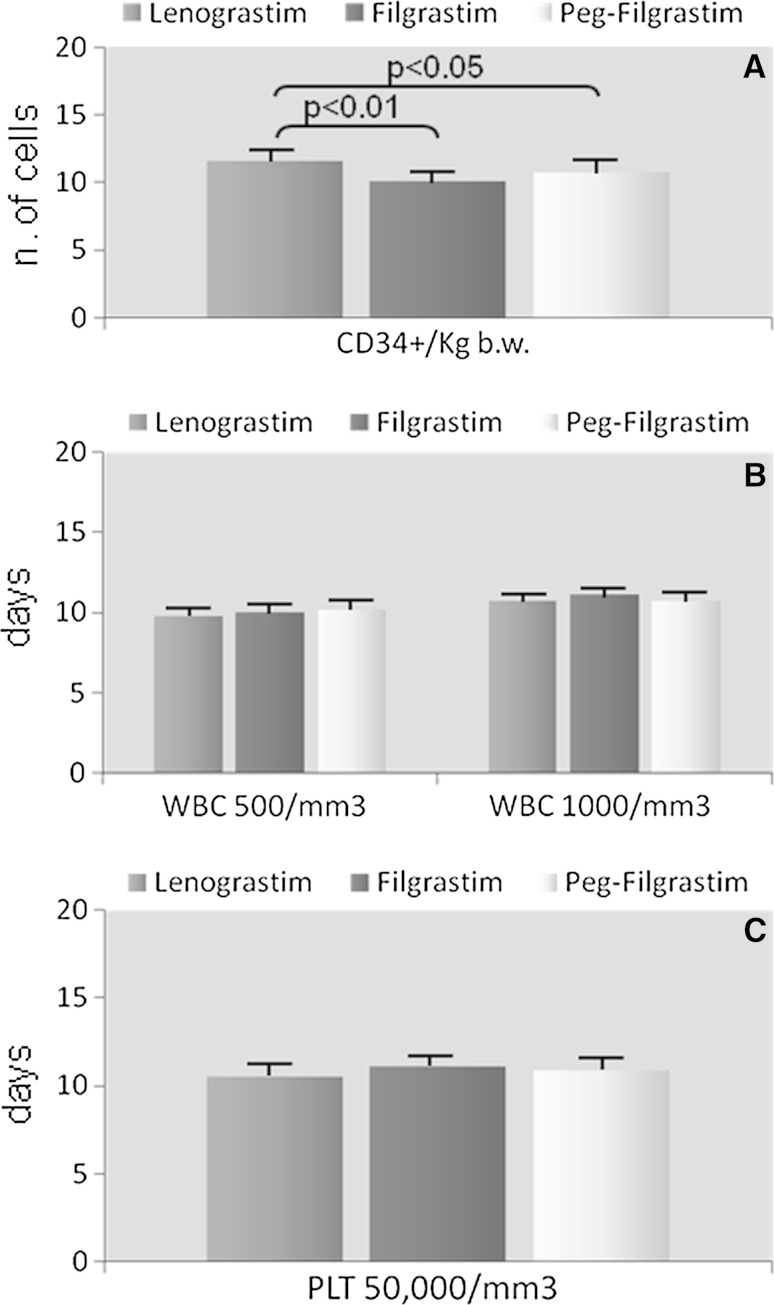
Collection of CD34+ cells (**a**) and differences observed in terms of days to WBC recovery ≥500 and ≥1,000/mm^3^ (**b**) and platelets ≥50,000/mm^3^ (**c**) in patients treated with lenograstim, filgrastim and pegfilgrastim

A significantly higher CD34+ collection was obtained from the patients in Arm A (11.6 ± 1.1 × 10^6^ CD34+/Kg bw) versus Arm B (10.04 ± 0.4 × 10^6^ CD34+/Kg bw) (*p* < 0.01) versus Arm C (10.76 ± 0.4 × 10^6^ CD34+/Kg bw) (*p* < 0.05) (Fig. 1a). The percentage of patients who reached the minimum collection target after two leukaphereses was higher in those treated with lenograstim (78 %) compared with those who were given filgrastim (48 %, *p* < 0.001) and pegfilgrastim (56 %, *p* < 0.005). Thus, glycosylated G-CSF provided more effective mobilization of bone marrow HPSCs.

No differences were observed among patients with MM, NHL or HL, nor according to the doses of cyclophosphamide (3, 4 or 7 g/m^2^) employed. More precisely, the following mobilization yields were achieved. Arm A patients: (a) with MM: 14.21 ± 2.8 × 10^6^ CD34+/kg bw; (b) with NHL: 16.14 ± 3.6 × 10^6^ CD34+/kg bw; and (c) with HL: 15.81 ± 3.8 × 10^6^ CD34+/kg bw (*p*: n.s.). Arm B: patients: (a) with MM: 10.82 ± 2.33 × 10^6^ CD34+/kg bw; (b) with NHL: 12.23 ± 1.9 × 10^6^ CD34+/kg bw; and (c) with HL: 12.84 ± 2.84 × 10^6^ CD34+/kg bw (p: n.s.); Arm C patients: (a) with MM: 11.82 ± 2.24 × 10^6^ CD34+/kg bw; (b) with NHL: 11.92 ± 3.3 × 10^6^ CD34+/kg bw; and (c) with HL: 13.36 ± 2.59 × 10^6^ CD34+/kg bw (*p*: n.s.).

The percentage of patients who reached the minimum collection target after two leukaphereses were 79 % in MM patients; 81 % in NHL patients and 78 % in patients with HL in Arm A (*p*: n.s.); 46, 48 e 48 % in Arm B (*p*: n.s.); 59, 61 e 54 % in Arm C (*p*: n.s.).

Finally, no significant differences were observed among patients with different lymphoma subtypes or with bone marrow involvement.

### Adverse events

Toxicity associated with the three regimens was similar in terms of bone pain, fatigue, fever, mucositis and infections (Table 2). All infections were treated with antibiotics and resolved following the recovery of a normal leucocyte count. Finally, no differences were observed within each group of patients in terms of days to WBC recovery ≥500 and ≥1,000/mm^3^ (Fig. 1b) and platelets to ≥50,000/mm^3^ (Fig. 1c). No platelet transfusions were needed.

**Table 2:** Adverse events

	Total adverse events	Treated with lenograstim	Treated with filgrastim	Treated with pegfilgrastim
Fever	12	6	3	3
Fatigue	5	2	1	2
Bone pain	7	3	2	2
Pseudomonas infection	2	0	1	1
Mucositis grade II	4	2	1	1
Staphylococcal infection	1	1	0	0
Fever of unknown origin	4	1	0	3
Nausea	9	2	4	3
Cough	2	2	0	0

## Discussion

High-dose chemotherapy followed by ASCT is considered the standard of care for young patients with newly diagnosed MM, in high-risk NHL and HL, in whom in several randomized studies [8, 12–14], this treatment has been shown to induce an increased rate of complete response, prolonged overall survival and reduced side effects compared with conventional chemotherapy.

Mobilization of HPSCs in cancer patients is a routine practice in high-dose therapy, followed by ASCT [8–10]. The mobilizing regimen usually consists of cyclophosphamide (3–7 g/mq) or disease-specific agents, in combination with an hematopoietic cytokine, usually G-CSF, which mobilizes HPSCs into the bloodstream, in particular when administered after myelosuppressive chemotherapy [12].

We analyzed differences in HPSCs mobilization in response to lenograstim, filgrastim or pegfilgrastim in patients with MM and lymphomas with the aim of determining which of the three G-CSFs was more effective in HPSCs mobilization.

In a previous study, we have already investigated the role of G-CSF glycosylation [15]. It modifies the chemical properties of G-CSF, including a higher molecular stiffness, pH, temperature and elastase resistance that result in a higher plasma half-life. Furthermore, glycosylation reduces the formation of aggregates and increases receptor affinity, causing an increase in bioavailability and molecular activity. Glycosylation of G-CSF is also associated with additional biological effects. For example, neutrophils exposed in vitro to non-glycosylated G-CSF exhibit reduced motility, morphological abnormalities, increased spontaneous actin polymerization and RhoA activation, a more immature phenotype and a slight reduction in the release of reactive oxygen species (ROS) compared with those exposed to glycosylated G-CSF. These features contribute to the impairment of all biological functions of these cells in response to external stimuli. Consistent with these findings, neutrophils exposed to non-glycosylated G-CSF may be less effective in preventing febrile episodes in patients with chemotherapy-related neutropenia when compared with those exposed to glycosylated G-CSF [16, 17].

Previous studies have shown that a lower dose of glycosylated G-CSF is as effective as the standard dose of non-glycosylated G-CSF for PBPC mobilization in patients undergoing ASCT. Furthermore, a more rapid mobilization has been observed in patients receiving lenograstim (median time to collection: 12 days) than in those receiving filgrastim (median time to collection: 13 days), which is in line with our results [18, 19].

The randomized trial conducted by the Italian group has shown a lower incidence of febrile episodes during the period of neutropenia in MM patients receiving lenograstim versus those administered filgrastim after high-dose cyclophosphamide for stem cell mobilization [1]. Patients treated with cyclophosphamide were randomly assigned to receive filgrastim or lenograstim. The lenograstim group presented not just a significantly higher absolute CD34+ cell number compared with the filgrastim group but also a less number of days (8 days against 9 of the arm B) needed to reach the target threshold of CD34+ cells, while no differences were detected in terms of collection efficacy. Pegfilgrastim results from the addition of a monomethoxy PEG to filgrastim, decreasing its serum clearance and markedly increasing its half-life. In fact, the G-CSF can be detected in the serum up to 14 days after a single administration of subcutaneous pegfilgrastim. Furthermore, in this way, its metabolism shifts totally to mature neutrophils. The growth factor would remain unchanged and active until the reconstitution of the number of circulating granulocytes.

Although pegfilgrastim is licensed for the prophylaxis of febrile neutropenia after cytotoxic chemotherapy, it is also an effective mobilizer of CD34+ cells, although not yet officially approved. In fact, pegfilgrastim compared favorably with the other G-CSFs after mobilizing chemotherapy for autologous HSC collection. The administration of pegfilgrastim following high-dose therapy and ASCT shortened the time to myeloid recovery when compared with conventional G-CSF. Plasma G-CSF levels were about 1 log higher with pegfilgrastim, but in the setting of autologous ASCT, this did not result into a faster hematopoietic recovery. Only few data are available on the biological effects of pegfilgrastim, which suggest that pegfilgrastim stimulation results in different functional properties of hematopoietic stem and progenitor cells compared with conventional G-CSF [20, 21].

Bassi et al. [22] compared the use of this type of growth factor with standard G-CSF in 64 patients with NHL using high-dose chemotherapy. At mobilization chemotherapy, the first 26 patients used unconjugated G-CSF, while the remaining 38 patients received pegfilgrastim. At the time of harvest, 25 patients collected stem cells after the use of G-CSF and 36 in the peg group. No statistically significant differences were observed in median peripheral CD34+ cells mobilized (77 vs. 71 μL) and in collected CD34+ cells (12.3 × 10^6^/kg vs. 9.4 × 10^6^/kg; *p* = 0.76). In the peg group, all patients collected the target CD34+ cells with a single apheresis with a greater proportion of “optimal” mobilizers (83 vs. 64 %; *p* = 0.05) showing that a single dose of pegfilgrastim could be a valid alternative to unconjugated G-CSF to mobilize CD34+ cells in lymphoma patients.

Our study shows that the glycosylated form of G-CSF provides the best results in the mobilization compared to non-glycosylated form and to pegylated form. This was evident both in terms of collection of target HPSCs and the number of leukaphereses required to achieve it, lower than the other two regimens. The patients included in the present study were well balanced between Arm A (glycosylated Hu G-CSF), Arm B (non-glycosylated rHu G-CSF) and Arm C (pegylated form), and there were no differences between groups that could affect the yield of HPSCs. There were no significant differences among the different diseases in terms of minimum number of CD34+ cells collected and the number of apheresis necessary to achieve the target, (4–6 × 10^6^ CD34+/Kg b.w.) and even among patients treated with 3, 4 or 7 g/m^2^ of cyclophosphamide. An average of two aphereses was sufficient both in patients with and without bone marrow involvement. Finally, radiotherapy did not affect the apheretic yield.

These findings indicate that, despite the three G-CSF are all safe in the mobilization procedure, lenograstim may represent the ideal partner of cyclophosphamide for mobilization of PBSCs in patients with lymphoproliferative disorders candidate to autologous transplantation.

Our report suffers from some limitations. The number of HL patients was not sufficient to determine any difference between the two G-CSF types in the disease. Also, patients’ accrual by a single institution cannot allow a phase III randomized study. However, center-to-center variations in the ASCT procedures prevented us to plan a multicenter phase III trial. Our study included three types of hematologic malignancies in order to reach a sufficiently large population to be examined. We did not monitor serum G-CSF levels or other parameters related to the mobilization process, which might have possibly revealed some clues for the lack of between-group differences. This kind of parameters, however, may be studied in a prospective randomized fashion.

Larger randomized trials are needed to confirm this conclusion and delineate the still unrevealed process of HPC mobilization by G-CSF following chemotherapy.
